# Supplementary material for: Structural Basis for the Inhibition of the Autophosphorylation Activity of HK853 by Luteolin
Source: Molecules. 2019 Mar 7;24(5):933. doi: 10.3390/molecules24050933 (PMC6429454; doi:10.3390/molecules24050933)
Supplement: Supplementary file 1 [file molecules-24-00933-s001.zip › Supplementary Materials/supplementary materials.pdf]

Supplementary Figure 1. Stability test (40 °C) of ATP and its degradation products. Peaks from 1 to 7 are AMP, Pi (free phosphorus), $^{31}P\gamma$ (ATP), $^{31}P\beta$ (ADP), $^{31}P\alpha$ (ADP), $^{31}P\alpha$ (ATP), and $^{31}P\beta$ (ATP), respectively. The ^{31}P spectra of ATP, ADP, and AMP are colored in red, blue, and orange for signal assignments, respectively. ATP, ADP, and AMP were all stable at 40 °C. The black spectrum shows the peaks of the reaction mixture (reaction conditions: 20 mM Tris, 50 mM KCl, 10 mM $MgCl_2$, and 8 mM ATP/ADP/AMP with 10% D_2O, pH 8.0).

Supplementary Figure 2. Lut inhibition of the autophosphorylation activity of EnvZ (n=3, *$p<0.01$, compared to no luteolin group). The ratio of ATP in the reaction mixture obtained by integration of ^{31}P NMR signals. No-luteolin group is indicated by ■. The luteolin-containing group is indicated by ▲, (Reaction conditions: 20 mM Tris, 50 mM KCl, 10 mM $MgCl_2$, 8 mM ATP, 0.4 mM EnvZ, and 0.8 mM Lut (in Lut group) with 10% D_2O, pH 8.0).

Supplementary Figure 3. Lut and ADP competition experiment. The spectrum in blue shows the ^{15}N-^{1}H HSQC signals of $HK853^{CA}$ binding with Lut, while ADP binding group in green (reaction conditions: 20 mM HEPES, 50 mM KCl, 10 mM $MgCl_2$, 0.4 mM $HK853^{CA}$, and 0.8 mM Lut/ADP with 10% D_2O, pH 7.0). The spectrum in red shows the ^{15}N-^{1}H HSQC signals of $HK853^{CA}$ when ADP competes with Lut. After $HK853^{CA}$ binding with Lut, the excess amount of ADP was added into the solution (reaction conditions: 20 mM HEPES, 50 mM KCl, 10 mM $MgCl_2$, 0.4 mM $HK853^{CA}$, 0.8 mM Lut, and 8 mM of ADP with 10% D_2O, pH 7.0).

Supplementary Figure 4. ^{15}N-^{1}H HSQC spectra of $HK853^{CA}$ in the presence of Api and Kae, respectively. (**a**) The spectrum in blue shows the ^{15}N-^{1}H HSQC signals of $HK853^{CA}$ without Api, while Api containing group is in red (reaction conditions: 20 mM HEPES, 50 mM KCl, 10 mM $MgCl_2$, 0.4 mM $HK853^{CA}$, and 0.8 mM Api with 10% D2O, pH 7.0).
(**b**) The spectrum in blue shows the ^{15}N-^{1}H HSQC signals of $HK853^{CA}$ without Kae, while Kae containing group is in red (reaction conditions: 20 mM HEPES, 50 mM KCl, 10 mM $MgCl_2$, 0.4 mM $HK853^{CA}$, and 0.8 mM Kae with 10% D_2O, pH 7.0).
