# Supplementary figures and images for: Structural Basis for the Inhibition of the Autophosphorylation Activity of HK853 by Luteolin
Source: Molecules. 2019 Mar 7;24(5):933. doi: 10.3390/molecules24050933 (PMC6429454; doi:10.3390/molecules24050933)

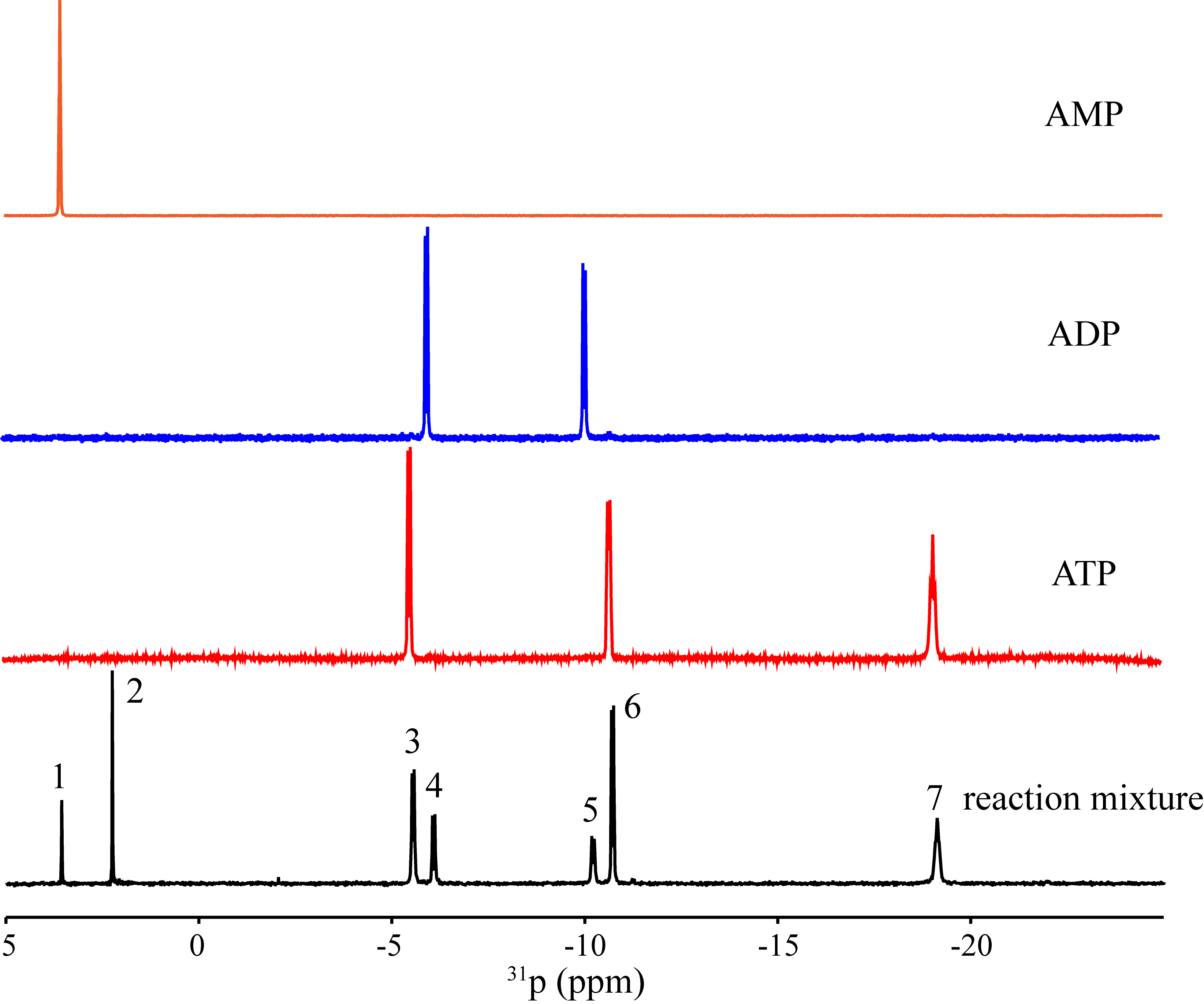

Supplement: Supplementary file 1 [file molecules-24-00933-s001.zip › Supplementary Materials/Figure S1.tif]

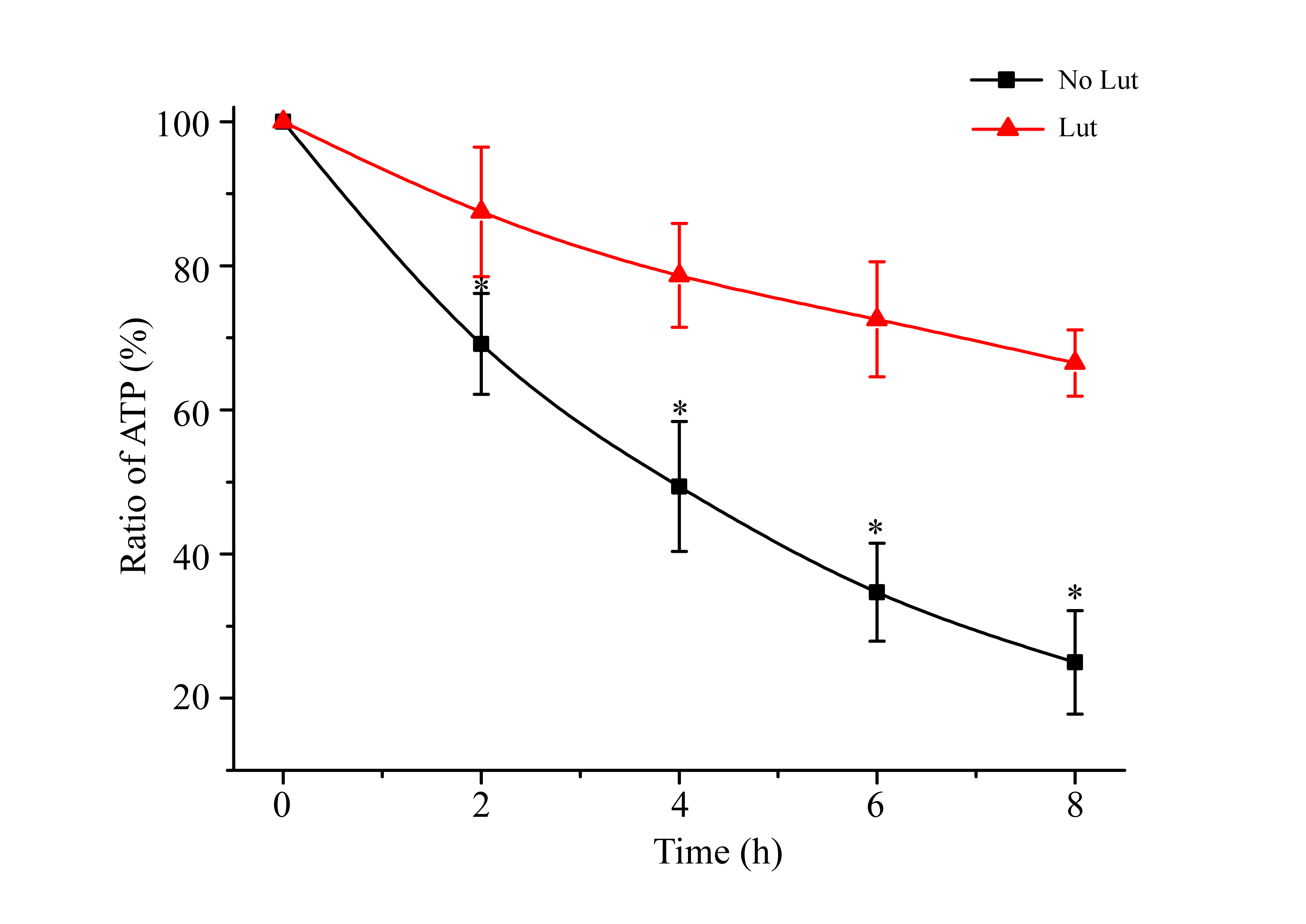

Supplement: Supplementary file 1 [file molecules-24-00933-s001.zip › Supplementary Materials/Figure S2.tif]

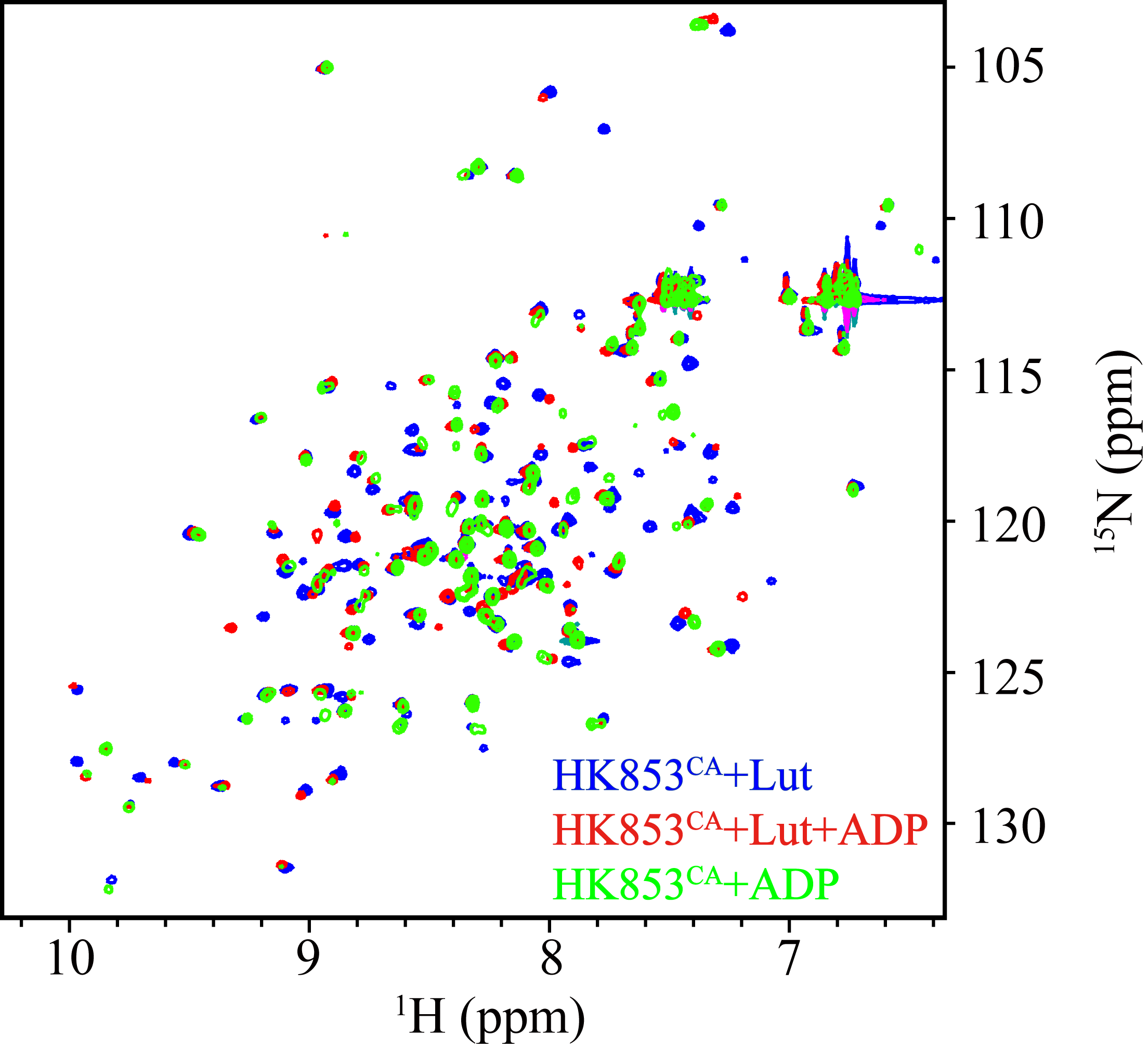

Supplement: Supplementary file 1 [file molecules-24-00933-s001.zip › Supplementary Materials/Figure S3.tif]

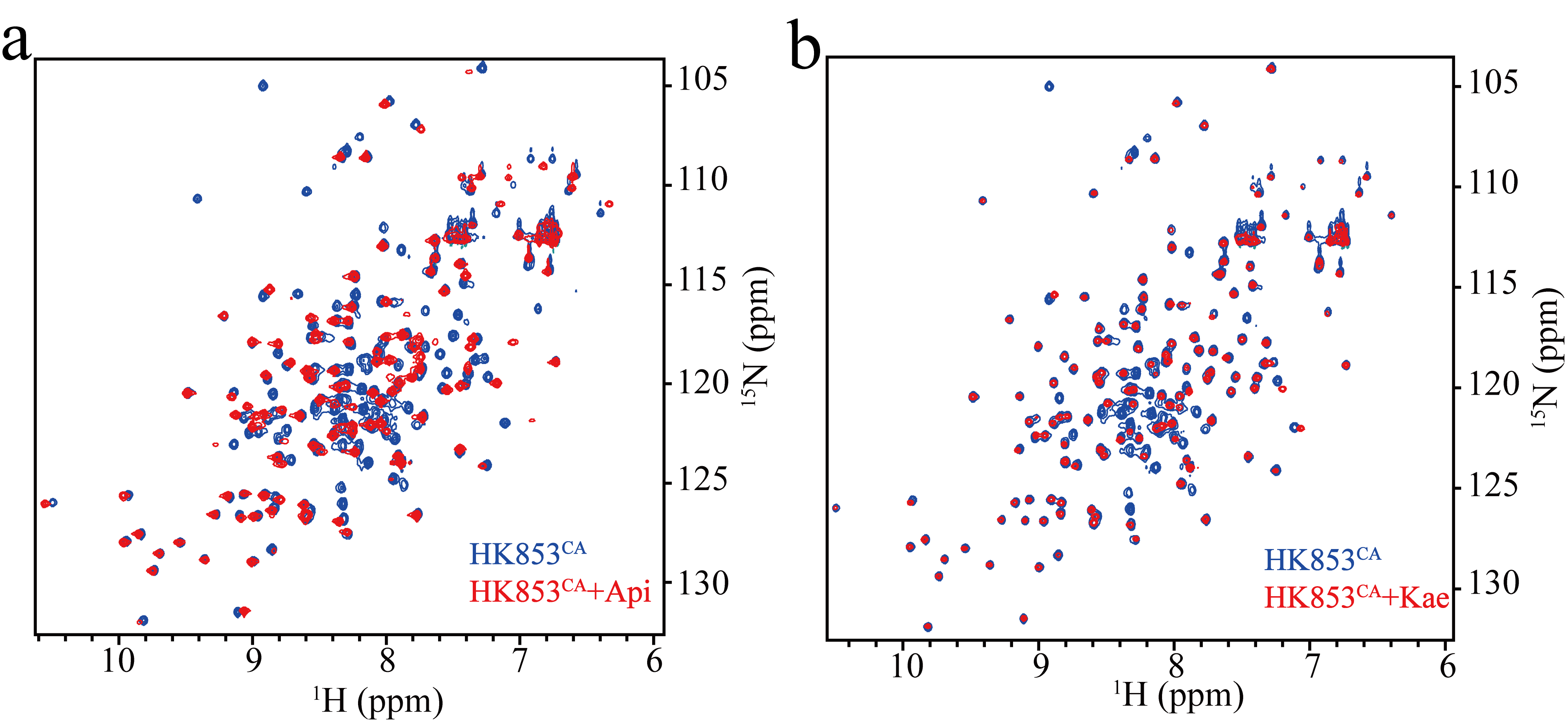

Supplement: Supplementary file 1 [file molecules-24-00933-s001.zip › Supplementary Materials/Figure S4.tif]
